# Incident Infection and Resistance Mutation Analysis of Dried Blood Spots Collected in a Field Study of HIV Risk Groups, 2007-2010

**DOI:** 10.1371/journal.pone.0159266

**Published:** 2016-07-14

**Authors:** Xierong Wei, Amanda J. Smith, David W. Forrest, Gabriel A. Cardenas, Dano W. Beck, Marlene LaLota, Lisa R. Metsch, Catlainn Sionean, S. Michele Owen, Jeffrey A. Johnson

**Affiliations:** 1 Division of HIV/AIDS Prevention, Centers for Disease Control and Prevention, Atlanta, Georgia, 30329, United States of America; 2 University of Miami, Miller School of Medicine, Miami, Florida, 33136, United States of America; 3 HIV/AIDS Section, Florida Department of Health, Tallahassee, Florida, 32399, United States of America; Institute of Infection and Global Health, UNITED KINGDOM

## Abstract

**Objective:**

To assess the utility of cost-effective dried blood spot (DBS) field sampling for incidence and drug resistance surveillance of persons at high risk for HIV infection.

**Methods:**

We evaluated DBS collected in 2007–2010 in non-clinical settings by finger-stick from HIV-positive heterosexuals at increased risk of HIV infection (n = 124), men who have sex with men (MSM, n = 110), and persons who inject drugs (PWID, n = 58). Relative proportions of recent-infection findings among risk groups were assessed at avidity index (AI) cutoffs of ≤25%, ≤30%, and ≤35%, corresponding to an infection mean duration of recency (MDR) of 220.6, 250.4, and 278.3 days, respectively. Drug resistance mutation prevalence was compared among the risk groups and avidity indices.

**Results:**

HIV antibody avidity testing of all self-reported ARV-naïve persons (n = 186) resulted in 9.7%, 11.3% and 14.0% with findings within the 221, 250, and 278-day MDRs, respectively. The proportion of ARV-naïve MSM, heterosexuals, and PWID reporting only one risk category who had findings below the suggested 30% AI was 23.1%, 6.9% and 3.6% (p<0.001), respectively. MSM had the highest prevalence of drug resistance and the only cases of transmitted multi-class resistance. Among the ARV-experienced, MSM had disproportionately more recent-infection results than did heterosexuals and PWID.

**Conclusions:**

The disproportionately higher recent-infection findings for MSM as compared to PWID and heterosexuals increased as the MDR window increased. Unreported ARV use might explain greater recent-infection findings and drug resistance in this MSM population. DBS demonstrated utility in expanded HIV testing; however, optimal field handling is key to accurate recent-infection estimates.

## Introduction

Community surveillance of persons at high risk of acquiring human immunodeficiency virus (HIV) infection is fundamental to evaluating the state of the HIV epidemic in local populations. There are several clinical and virologic measures useful for evaluating potential drivers of the epidemic in different demographic populations and risk behaviors. Enhancing surveillance beyond rudimentary serostatus observations can add a great deal to our understanding of risk behavior and transmission potential and help to focus prevention efforts where they might have the greatest impact. To that end, expanding testing of biologic specimens from surveilled HIV-positive persons would augment often limited surveillance data.

Two significant factors limiting expanded testing for surveillance are additional costs for acquiring specimens and access to marginalized populations who may not be regularly engaged in clinical care. Because HIV epidemics are local and reflect community perceptions and practices, virologic data, which is often lacking particularly from the highest risk communities, can help to uncover epidemiologic associations that might otherwise be difficult to realize. To help address deficiency issues, cost-effective methods can be designed to be favorable for outreach testing in various settings. An attractive specimen type amenable to collection in non-clinical settings is the dried blood spot (DBS) which can be obtained from a simple fingerstick and spotting onto a card. DBS alleviate the expense of phlebotomy supplies and cold chain storage, and have a history of success in infant genetic and serology testing [1–3]. While there are limitations to DBS, such as volume of material and limited validation with commercial diagnostic kits, antibody stability and the presence of viral RNA, DNA, and proteins support this specimen type both for immunologic and genetic testing in surveillance.

Whereas HIV seroprevalence in a demographic population is an important indicator of potential HIV exposure risk within that population, the recency of infection is a key measure of how many new infections are occurring. Therefore, an accurate measure of recent infections is vital for gauging ongoing HIV transmission and any impact that prevention efforts might have on reducing HIV acquisition. A useful immune property for testing infection recency is antibody avidity which measures how strongly an HIV-infected individual’s antibodies bind to HIV proteins. During infection, extended time in the presence of viral antigen stimulation is required for the humoral response to select and expand avid antibody clones. This immune evolution can be exploited to establish within a population a binding strength cutoff, or avidity index (AI), for determining recent infection. Ideally, the AI would not be sensitive to subsequent decreases in viral antigen stimulation and AIDS-stage disease progression.

Surveillance of HIV mutations that confer resistance to antiretroviral drugs (ARVs) can convey many traits about the infected population being surveyed. Drug resistance mutations may arise in persons on therapy if the ARV medications are not taken as prescribed. Those individuals may transmit drug-resistant viruses to others if risk behavior prevention practices are not followed. Furthermore, the prevalence of drug resistance in a community is related to the proportion of people who have accessed ARVs [4], presumably through engagement in care. Thus, along with other surveillance data, viral amplification and drug resistance prevalence are biologic markers that gives an impression of a community’s access to care, adherence to care, and, with particular resistance mutation transmission, information on risk behavior in persons who may be aware of their status.

In the pilot study presented here, we report on expanded testing of DBS collected anonymously in non-clinical settings during 2007–2010 from persons at high risk for acquiring HIV infection. The DBS collection was part of CDC National HIV Behavioral Surveillance (NHBS) in Miami, Florida (USA) and included engaging those who may not have otherwise sought HIV testing or may have lapsed from care. The objective was to evaluate whether DBS collected by local public health personnel in field settings might support recency testing and sensitive drug resistance screening as examples of enhanced testing to complement behavioral data collected in the survey. We show the findings provided a richer depiction of HIV infection and uncovered unique immunologic and virologic differences among the risk groups evaluated.

## Methods

### Study Populations

CDC National HIV Behavioral Surveillance (NHBS) conducts annual cross-sectional interviews and HIV testing in metropolitan statistical areas (MSAs) with high AIDS prevalence to monitor trends in HIV-associated risk behaviors, HIV testing, use of HIV prevention services, and HIV infection among populations at risk for acquiring HIV [5]. Populations considered at increased risk of acquiring HIV infection are sampled in annual rotating cycles among men who have sex with men (MSM), persons who inject drugs (PWID), and heterosexuals at increased risk of HIV infection [5]. Either venue-based time-space sampling or respondent-driven sampling methods are used to recruit individuals fitting the eligibility criteria for each population sampled [6–9]. Behavioral data are collected using a standardized questionnaire during anonymous face-to-face interviews with trained staff members. All participants interviewed are offered anonymous HIV tests.

In one project site, since 2007, HIV testing has been performed by collecting finger stick blood specimens for rapid testing in the field followed by DBS laboratory-based confirmation of preliminary positive results by Western blot. After this initial testing by a local laboratory, the remaining DBS collected between 2007 and 2010 were stored at -70°C for one to four years prior to shipping on dry ice to the CDC for avidity and resistance testing. DBS tested for this analysis were collected from participants who tested positive for HIV infection in two heterosexual cycles (n = 124; 50% female) and one MSM (n = 110) and PWID (n = 58; 33% female) cycle in the Miami/Ft. Lauderdale, Florida MSA from 2007 to 2010. The overall HIV prevalence for all participants surveyed who consented to testing was 23.8% for MSM, 16.5% for PWID, and 8.1% and 8.0% for the two heterosexual cycles. Within each population there were participants who reported co-risk behavior, such as MSM who also reported PWID activity or heterosexual males who also reported MSM contacts. Specimens from participants reporting more than one risk were analyzed separately from those reporting single risk; both because we cannot ascertain which behavior may have led to their infection, and also to assess if recency or resistance findings differed between persons reporting single or multiple risks. Only those participants reporting MSM contact or drug injection in the 12 months prior to survey were considered in the assessment of co-risk. The proportion of all persons surveyed in each cycle who reported ever having antiretroviral drug (ARV) exposure was 2.8% for heterosexuals, 7.1% for PWID, and 9.2% for MSM. Greater than 99% of all participants interviewed in Miami/Ft. Lauderdale between 2007 and 2010 consented to finger stick for HIV testing as well as storage and future testing of their blood specimen. The CDC IRB reviewed the procedures for participant anonymity and consent forms and approved this testing evaluation under the determination that it did not involve identifiable human research and participants consented to future testing.

### Recency Testing

Specimens were tested at the CDC using an avidity-based modified GS^™^ 1/2 plus O-based (Bio-Rad) assay [10]. Briefly, the assay measures an avidity index (AI), a ratio of optical densities from paired immunoassay wells, one treated with 0.1 M DEA and one with wash buffer. Higher AI ratios likely indicate long-term infections due to more avid antibodies that are less affected by the DEA treatment. A protocol for avidity testing of DBS was developed in the CDC laboratory and validated against 45 archived DBS specimens from Cameroon cryopreserved for seven years and five recently prepared DBS specimens from the US, both sets were prepared in laboratory settings and stored frozen in the presence of desiccant and humidity indicators. For the DBS in-house protocol assessment, various elution volumes with specimen diluent provided in the kit, as well as different elution periods and incubation temperatures were examined. The AIs from the paired DBS and plasma testing were compared by linear regression analysis. The elution conditions that yielded results which best matched those of plasma was the elution protocol selected. In evaluating DBS sampling for avidity testing, the avidity results from complete spots (~50 uL blood) and six-millimeter diameter punches (~13 uL blood) were each compared to avidity results from 1:10-diluted matched plasma specimens. The punch sampling was to assess performance of a smaller sample size for when specimen availability is limited.

The optimized protocol selected was elution of the spot or punch overnight in 300 uL kit buffer at 4°C after which 100 uL of eluate is applied to the microtiter wells. The linear regressions of the AI results for plasma compared to matched complete spot and one-punch samples were determined (see S1 Fig).

To evaluate whether the choice of AI cutoff within the linear range of increasing ratios over time affects relative recency results between risk populations, three AI cutoffs were evaluated, 25% (0.25), 30% (0.30), and 35% (0.35). For subtype B viruses, the conservative AI of 25% represented an MDR of 220.6 days (95% confidence limit: 187.2, 253.9 days), the 30% AI represented an MDR of 250.4 days (95% confidence limit: 215.6, 285.2 days), and the 35% AI represented an MDR of 278.3 days (95% confidence limit: 236.2, 320.4 days), as evaluated with plasma and using an incidence parameter of T = 2 years and logit generalized additive modelling (GAM). Employing longer recency periods would allow for greater inclusion of recent infections, but may also be subject to a higher false-recent rate (FRR) where established infection reactivity increasingly falls within the recency cutoff. Specimens that produced AI results between 30%-50% were repeat tested (the mean of duplicate sample repeats were considered to be the final AI) to verify that the value was above or below 30%. For the evaluated matched sample set described above, using the recommended AI cutoff of <30% (manuscript in preparation) found an increasing recency trend with smaller specimen size in that 17.8%, 22.2%, and 24.4% of plasma, entire spot, and one-punch samples, respectively, provided results under the AI cutoff value. Because some cards collected in the South Florida surveys had less available sample, all field-collected DBS of the study populations in this report were tested using one 6 mm punch for consistency. For calculating population incidence estimates we included the HIV-negative participants surveyed in the denominator.

Because it is known that ARV use can diminish anti-HIV immune responses, participants who divulged ARV exposure were analyzed for infection recency separately from those who reported to be ARV-naïve. Thus, recent infection findings in the ARV-exposed population can help to determine the contribution of ARV use to the FRR.

### Drug Resistance Screening

From the same DBS cards as was used for avidity testing, one complete spot, approximately 50 uL of whole blood, was extracted manually using NucliSens (bioMerieux) silica method and both total nucleic acids and DNase-digested extracts were amplified in PCRs with and without reverse transcriptase [11]. Amplified products were screened with sensitive, rapid real-time PCR assays [12,13] for reverse transcriptase mutations M41L, K65R, K103N, Y181C, M184V, and T215Y/revertants (mutation frequency cutoffs of 0.3–1%) as sentinel markers for assessing transmitted and acquired drug resistance. Mutations M41L, K103N, Y181C, and 215Y represent some of the more common transmitted resistance mutations, whereas, K65R and M184V are rarer in prevalence but important mutations to the commonly prescribed antiretrovirals, tenofovir and emtricitabine, which are also under consideration as pre-exposure prophylaxis for the prevention of HIV transmission. In the resistance analyses, mutation linkage [14] and DNA/RNA compartments were evaluated for archived latent and expressed variants, the latter is also important for assessing risk of transmission. Similar to recency testing, participants reporting ARV use were analyzed separately from those who reported being ARV-naïve, with resistance mutations in the latter group interpreted as transmitted drug resistance.

## Results

### HIV-positive self-reported demographics

A total of 292 DBS specimens from HIV-positive participants were evaluated. Of the 124 heterosexual survey cycle participants, 10 (8%) reported MSM contact, five (4%) reported as PWID, and 49 (40%) reported having used ARVs (Table 1). The heterosexual survey participants were predominantly Black (86%) and evenly male/female (50% each) with a median age of 43 years. Of the 110 participants from the MSM cycle, one reported engaging in PWID risk behavior and 44 (40%) reported ARV experience. The MSM cycle participants were predominantly Hispanic/Latino (64%) with a median age of 37 years. Of the 58 HIV-positive participants of the PWID cycle, 11 (19%) also reported MSM contact and 23 (40%) reported having used ARVs. The majority of PWID were Black (71%) and male (67%) with a median age of 50. Age was not significantly different among the survey cycles (F-test).

**Table 1:** HIV-positive participants with DBS available for testing.

	HET n (%)	MSM n (%)	PWID n (%)
Total	124	110	58
White*	4 (3)	13 (12)	7 (12)
Black	107 (86)	23 (21)	41 (71)
Hispanic/Latino	12 (10)	70 (64)	9 (16)
Female	62 (50)	N/A	19 (33)
ARV-experienced	49 (40)	44 (40)	23 (40)
MSM co-risk^Δ^	10 (8)	N/A	11 (19)
PWID co-risk^Δ^	5 (4)	1 (0.9)	N/A

* Ethnicity of few mixed/other races not shown;

^Δ^ includes ARV-naïve and experienced; ARV, antiretroviral drug; N/A, not applicable

### Avidity recent-infection reactivity

#### Heterosexuals

Five (6.9%) of 72 specimens from ARV-naïve, HIV-infected heterosexual participants reporting no other risk factors for HIV recorded an AI of <25%, and there were no additional findings when the AI cutoff was increased to 30%. However, at a 35% AI, recent infection findings increased by three more individuals to 11.1%. None of the heterosexuals with recency AI values <30% was aware of their infection status. When including 13 participants with MSM or PWID co-risk behaviors in the analysis, the proportion with AI reactivity at <30% AI went from 6.9% to 8.3%; the increase in recent-infection results involved one each with PWID and MSM co-risk. Of 37 ARV-experienced heterosexuals, one (2.7%) recorded an AI <30%; this was a participant who reported having a non-reactive HIV test 20 months prior to interview. The remainder of the ARV-experienced heterosexuals recorded AIs of >50%.

#### MSM

Twelve (18.5%) of 65 ARV-naïve participants who reported being MSM as the only HIV risk had AI results <25%, and three more (23.1%) were identified at a cutoff of <30%. At a 35% AI cutoff, the number of MSM recording as recent increased to 17 (26.2%) (Table 2). One ARV-naïve MSM participant also reported PWID activity and had an AI that was consistent with a long-term infection. Of the 44 ARV-exposed MSM, 7 were found to have an AI <30%, suggesting a false recent infection finding in 15.9% of ARV-treated MSM cases. All seven were believed to have been infected for more than two years based on self-reported testing history. For ARV-exposed participants, the proportion with results below the 30% AI cutoff were 6-times higher for MSM (15.9%) than for heterosexuals (2.7%) (P = 0.06, Fisher’s exact).

**Table 2:** Recency test results using, 25%, 30% and 35% avidity indices.

Survey populations	ARV status and HIV co-risk	AI < 25%n/total (%)	AI < 30%n/total (%)	AI < 35%n/total (%)
Heterosexual				
	ARV-naive^†^	5/72 (6.9)	5/72 (6.9)	8/72 (11.1)
	MSM co-risk	0/9 (0)	1/9 (11.1)	2/9 (22.2)
	PWID co-risk	1/4 (25)	1/4 (25)	1/4 (25)
	ARV-experienced^†^	1/37 (2.7)	1/37 (2.7)	1/37 (2.7)
MSM				
	ARV-naive^†^	12/65 (18.5)	15/65 (23.1)	17/65 (26.2)
	PWID co-risk	0/1 (0)	0/1 (0)	0/1 (0)
	ARV-experienced^†^	6/44 (13.6)	7/44 (15.9)	8/44 (18.2)
PWID				
	ARV-naive^†^	1/28 (3.6)	1/28 (3.6)	1/28 (3.6)
	MSM co-risk	0/7 (0)	0/7 (0)	0/7 (0)
	ARV-experienced^†^	1/19 (5.3)	1/19 (5.3)	1/19 (5.3)

^†^ Reported only single risk for survey cycle

#### PWID

Only one (3.6%) of 28 samples from ARV-naïve PWID who reported injection practices as the only HIV risk recorded an AI <25%, and no additional PWID participants reacted as recent when increasing the AI to <35%. The recent-infection PWID was aware of their infection status. The number with recent-reactivity test results at <30% AI did not change when including the 7 PWID who also reported MSM behavior. Of the 19 ARV-exposed PWID, one (5.3%) produced an AI <25%; this individual reported having a negative HIV test 20 months prior to interview. The remainder of the ARV-experienced PWID, including four who also had MSM contacts, recorded AIs >40%.

At the conservative 25% AI cutoff, the proportion of ARV-naïve HIV-positive MSM who were recent-infection reactive was 5.1-times higher than what was observed for HIV-positive PWID (p = 0.10, Fisher’s exact test) and 2.8-times higher than HIV-positive heterosexuals (p = 0.067). At the 30% cutoff, recent-infection reactivity for MSM was 6.4-times that of PWID and 3.3-times heterosexuals (MSM vs PWID p = 0.033; MSM vs heterosexuals p = 0.014). And at the 35% cutoff, recent-infection reactivity for MSM was 7.3-times that of PWID and 2.4-times heterosexuals (MSM vs PWID p = 0.010; MSM vs heterosexuals p = 0.028); the disproportionate increases achieving significance due to a greater accumulation of endpoints.

Comparing recent-infection findings as a proportion of all ARV-naïve participants (HIV-positive and –negative) in the populations surveyed, we found that at the 25% AI the percent MSM with reactivity below the cutoff (2.33%) was 8-times that of heterosexuals (0.29%) (Fisher’s exact p<0.0001) and 10.6-times that of PWIDs (0.22%) (p = 0.0038). At the 30% cutoff, the proportion of MSM with reactivity <30% (2.92%) was 10.1-times that of all heterosexuals (0.29%) (p<0.0001) and 13.3-times that of PWIDs (0.22%) (p = 0.0006). And at the 35% AI cutoff, the proportion of MSM with recent-infection findings (3.31%) was 7.2-times that of all heterosexuals (0.46%) (p<0.0001) and 15-times that of PWIDs (0.22%) (p = 0.0002). Therefore, among all persons included in each survey cycle, the disproportion of MSM with recent-reactive findings relative to heterosexuals and PWID was 26% greater at the 30% AI cutoff that at a 25% cutoff.

### Transmitted and ARV-experienced drug resistance

Antiretroviral drug resistance was assessed separately for persons self-reported as being ARV-naïve versus ARV-exposed. Nucleic acid recovery from specimens of ARV-naïve participants varied among the three cycles, with the greatest success in amplification for MSM (86%) and less for PWID (70%) and heterosexuals (68%) (Table 3). After recency testing, some blood cards did not have sufficient sample available for resistance testing and those participants were not included in the nucleic acid analyses.

**Table 3:** Drug resistance mutation testing of DBS specimens.

Survey cycle	HIV nucleic acid analysis	ARV-naïve	ARV-experienced
Heterosexual			
	Amplifiable NA, n/total (%)	13/19 (68)	14/32 (44)
	Resistance detected, n/amplifiable (%)	3^a^/13 (23)	4/14 (29)
	Resistant variants identified (% of amplifiable specimens)	103N (23)	103N (7) 103N/184V (7) 184V (7) 181C (7)
MSM			
	Amplifiable NA, n/total (%)	55/64 (86)	29/41 (71)
	Resistance detected, n/amplifiable (%)	15/55 (27)	9/29 (31)
	Resistant variants identified (% of amplifiable specimens)	103N (16) 103N/215Y^ (2) 181C (2) 184V (2) 181C/184V (6)	103N (36) 184V/190A/215Y (3)
PWID			
	Amplifiable NA, n/total (%)	7/10 (70)	12/37 (32)
	Resistance detected, n/amplifiable (%)	2^b^/7 (29)	3^b^/12 (25)
	Resistant variants identified (% of amplifiable specimens)	103N (14) 103N/181C (14)	103N (8) 184V (17)

NA, HIV nucleic acids;

^a^ one reported also PWID risk;

^b^ one reported also MSM contact;

^ includes 215 revertants H, N, L, D, or E.

Thirteen of 19 specimens from ARV-naïve heterosexuals had amplifiable virus of which real-time PCR screening identified three (23%) with drug resistance. Only one of the three had an AI <30% and all involved only the K103N mutation. ARV experience was reported by 32 heterosexual participants for whom samples were available and 14 (44%) had amplifiable HIV nucleic acid. Four of the 14 (29%) with amplifiable virus had drug resistance mutations, including one reported MSM contact, who had mutations to two drug classes. (Table 3). This implied 13% of ARV-experienced heterosexuals had detectable drug resistance.

Resistance mutations were identified in 15 (27%) of 55 ARV-naïve HIV-positive MSM with amplifiable viral nucleic acids. Of the 55 amplifiable spots, 12 were from persons who recorded AIs <30%, of who two (17%) had resistance (Table 3). In this group of MSM reporting no ARV exposure, infections with multiple (≥3) resistance mutations were detected only in persons who knew of their infection. The RT mutations detected in ARV-naïve MSM were 10 K103N, 4 Y181C, 4 M184V, and two mutations at codon 215. Of 41 ARV-experienced MSM, 29 (71%) had amplifiable virus sequence. Nine of the 29 (31%) ARV-experienced with amplifiable virus had resistance mutations. DNase pre-treatment found that 7 of the 9 had amplifiable RNA and with RT-minus reactions the remaining two had mutations in only the proviral DNA compartment. This implied that 17% of HIV-positive MSM reporting ARV use were *expressing* drug-resistant virus at a level that was sufficiently high to be detectable in DBS extracts equivalent to ~0.005 mL (>200 copies/mL) of plasma. In ~10% of amplified HIV-positive MSM samples, B/C recombinant subtypes were identified with high similarity to pol sequences (GenBank loci AY999571, AY390178, JF487836) from Brazil, suggesting an epidemiologic link between the HIV population in that country and the predominantly Hispanic/Latino MSM in Miami.

Seven (70%) of 10 blood spot specimens from ARV-naïve PWID had amplifiable HIV nucleic acid of who two (29%) had (K103N and/or Y181C) drug resistance; both resistance mutations are known to be stable and transmissible over sequential infections (Table 3). One of the two with resistance had a recency test AI <30%, the second, with established infection, reported also having MSM contact and never obtained previous test results. Twelve (32%) of the 37 ARV-experienced PWID had amplifiable virus of whom three (25%), all with AIs >60%, had resistance mutations. One of the three with resistance also reported MSM contact. In all, 8% of the ARV-experienced PWID had detectable drug resistance mutations.

## Discussion

We report on population-specific findings from recency testing and drug resistance screening in assessing the suitability of field-collected DBS for expanding HIV testing in surveillance. The immunologic and nucleic acid test evaluations involved samples that spanned four years of rotating surveillance cycles of men who have sex with men (MSM), persons who inject drugs (PWID), and heterosexuals at increased risk for HIV infection. We previously described the utility of high-integrity laboratory-prepared DBS for HIV testing [7] and demonstrated very good correlation in AI values derived from laboratory-prepared DBS and matched plasma specimens. The AI cutoff of 30%, as compared to 25% or 35%, was determined to be optimal for subtype B plasma samples by analyzing known seroconversion cohorts, with the aim of minimizing the false recent rate and maintaining a sufficiently long MDR (manuscript in preparation). However, defining wider windows of recency may also allow for greater misclassification of recent infections with samples of poorer integrity. Because of the uncertainty of the integrity of field-collected DBS in the present study, we did not want to compare population-specific findings using a single biologic cutoff. Therefore, we evaluated recency test results using three cutoffs over an AI range of 25–35% which corresponded to an MDR window that spanned ~57 days.

For each surveillance population sampled, recency test results and drug resistance were evaluated first for persons who indicated engaging in only the single risk behavior of interest for each surveillance cycle. We found that a greater proportion of MSM produced results below the recency assay cutoff than did PWID and heterosexuals, and that this disproportion was greater at the 30% AI cutoff than at the 25% cutoff. The disproportionate increase in recent findings for MSM at the wider MDR relative to the other populations may reflect a truly higher infection incidence in MSM, but that cannot be ascertained appropriately from this study. Subsequent to the single-risk evaluation, persons reporting additional risk behaviors were then included in the analyses to assess whether the additional risk(s) had an impact on the frequency of recent infection findings. The number of recent-infection findings in the PWID population did not increase when including in the analysis those who also reported contact with MSM, who have a higher HIV prevalence in this community. The absence of an increase in recent infection when adding MSM contact suggested that this PWID population consisted of relatively established infections. However, the inclusion of MSM and PWID co-risk in the heterosexual population analyses did disproportionately increase the number of cases below the avidity cutoff compared to heterosexuals who reported no other risks, revealing a potential influence of MSM and PWID activity on HIV acquisition assessments in heterosexual surveys.

From the recency testing of ARV-experienced participants emerged a peculiar finding in that a much higher proportion of (false) recent-infection results were obtained with MSM currently/formerly taking antiretroviral drugs as compared to ARV-experienced heterosexuals and PWID. As to why ARV exposure appeared to be associated with greater false-recent results for the MSM population in particular is unknown. One possibility is that a larger proportion of MSM initiate ARV therapy earlier after infection relative to PWID and heterosexuals. Another possibility is that ARV off-label use may be more prevalent in the MSM population, which may lead to more cases of seroconversion in a setting of residual antiviral activity and, therefore, decreased antibody avidity. Regardless, with self-reporting it is not possible to confirm in what context ARVs may have been used. Of note, both the heterosexual and PWID ARV-experienced participants with avidity test results below the 30% cutoff were reported to have been infected for less than 20 months and did not have amplifiable virus, suggesting that they began therapy early in the course of their infections and maintained virologic suppression. The circumstance that both individuals had initiated suppressive therapy relatively early in infection is in agreement with what is known of early therapy associating with lower avidity ratios. Conversely, the ARV-experienced MSM with results below the AI cutoff all reported being infected for more than two years and all had detectable viral RNA. The finding that a few of the MSM had the K103N resistance mutation does not alone substantiate a history of ARV use as this is a commonly transmitted resistance mutation. Nonetheless, fewer ARV-experienced MSM were suppressing their infections and proportionally more were recording as recent infections as compared to ARV-experienced PWID and heterosexuals. Additionally, consistent with the reported greater prevalence of ARV experience in MSM relative to the other populations, transmitted drug resistance mutations were more common in ARV-naïve MSM than in heterosexuals and PWID.

The success of nucleic acid amplification from this DBS field sampling of ARV-naïve, HIV-infected populations was less than the >94% success historically obtained from fresh or cold-stored laboratory-prepared DBS [11, 12] (and unpublished data) implying that a portion of samples from all cycles were compromised. However, this was not entirely unexpected as at the time of survey the DBS field collection process was not optimized for nucleic acid preservation. Nevertheless, there were notable differences in viral nucleic acid recovery between survey cycles in that amplification was more successful for the MSM samples than for heterosexuals and PWID. This lends to the possibility of an overestimation of recent-infection findings for the heterosexual and PWID populations due to a greater prevalence of lower quality specimens. Importantly, this also revealed that the higher prevalence of recent-infection results for MSM was not due to poorer sample quality relative to the other populations.

There are several limitations to this pilot evaluation of field-collected DBS for expanded HIV surveillance testing. Foremost, handling time and environmental conditions prior to specimen freezing likely had an effect on sample integrity. With the possibility that nucleic acid degradation occurred, amplifiable RNA and transmitted drug resistance may have been underestimated in the ARV-naïve populations, and the degree of virus suppression may have been overestimated for ARV-experienced cases. The analysis of transmitted drug resistance was also restricted by the high proportion of participants reporting ARV experience. For recency testing, as mentioned above, a loss of sample integrity or unreported ARV use may erroneously increase the proportion of individuals with reactivity below the AI recency cutoff. Moreover, the small, single-punch specimen evaluated due to limited sample availability could have resulted in higher recency findings across all populations; however, any overall higher FRR as a result of this should not have affected relative indices in the comparisons. The DBS in the present study were all collected at one site and may not be representative of sites with less experience in DBS collection or that have higher ambient temperature and humidity conditions which may yield lower integrity specimens. Similarly, owing to the sampling strategies for NHBS [4–6], the MSM, PWID, and heterosexuals who provided specimens for this study may not be representative of other MSM, PWID, and heterosexuals in the Miami / Fort Lauderdale area. Last, as with any self-reported data, risk behavior and ARV use data are subject to recall error and social desirability bias.

In conclusion, the expanded testing of remnant DBS presented here provided additional biologic and virologic information to augment the HIV behavioral and diagnostic test data for the different high-risk populations surveyed. A high proportion of NHBS participants at this site accepted finger sticks, supporting that blood sampling in non-clinical settings can be practicable. We found that residual field-collected blood spots could be used for recency and sensitive drug resistance testing and the results were in accord with historical relative trends in HIV acquisition for each risk population. Although DBS collected in non-clinical settings can be a cost-effective and simple approach for enhanced outreach testing and surveillance, specimen preparation and handling are key to accurate test results. The findings support using optimized DBS processing and collection for field settings to expand reach to populations at high risk for acquiring HIV.

## Supporting Information

S1 Fig Correlation of avidity indices from 45 plasma samples with (A.) entire spots and (B.) single 6-mm punches from matched DBS. (PDF)
