# Supplementary figures and images for: Incident Infection and Resistance Mutation Analysis of Dried Blood Spots Collected in a Field Study of HIV Risk Groups, 2007-2010
Source: PLoS One. 2016 Jul 14;11(7):e0159266. doi: 10.1371/journal.pone.0159266 (PMC4944983; doi:10.1371/journal.pone.0159266)

A.

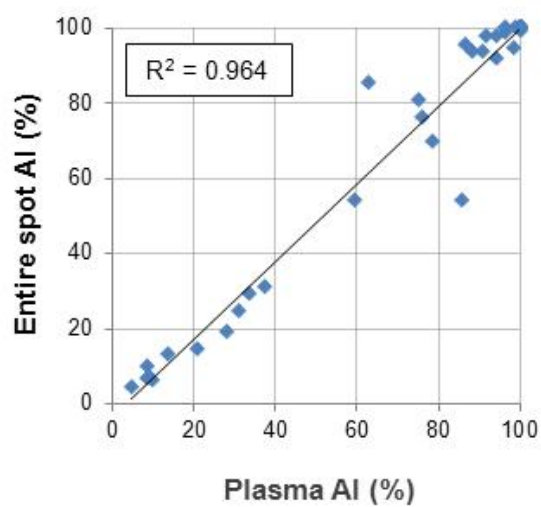

B.

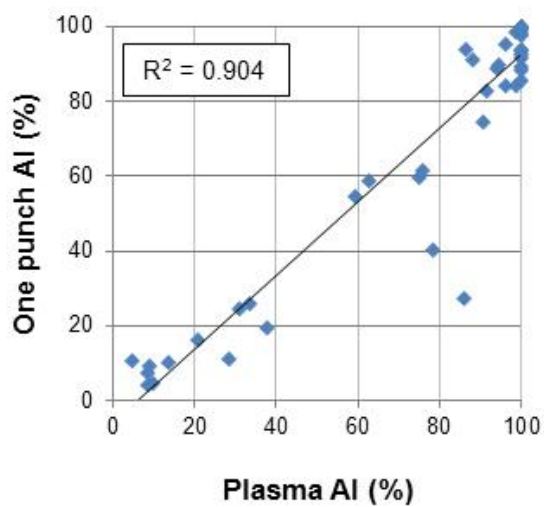

Supplement: S1 Fig — (PDF) [file pone.0159266.s001.pdf]
